# Supplementary material for: Elongator and codon bias regulate protein levels in mammalian peripheral neurons
Source: Nat Commun. 2018 Mar 1;9:889. doi: 10.1038/s41467-018-03221-z (PMC5832791; doi:10.1038/s41467-018-03221-z)
Supplement: Supplementary file 1 — Supplementary Information [file 41467_2018_3221_MOESM1_ESM.pdf]

**Elongator and codon bias regulate protein levels in mammalian peripheral neurons**

Goffena et al.

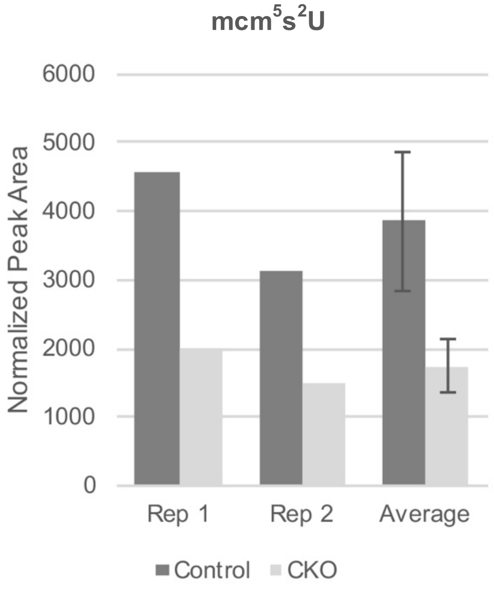


**Supplementary Figure 1**. Liquid chromatography-mass spectrometry (LC-MS) based tRNA modification analysis shows that $mcm^5s^2$-modified uridine levels are depleted in mammalian neurons in the absence of Elongator. DRG were collected and pooled from 4 control and 4 CKO embryos per biological replicate. Peak area values of $mcm^5s^2U$ are shown normalized to the quantity of purified tRNA for each sample.

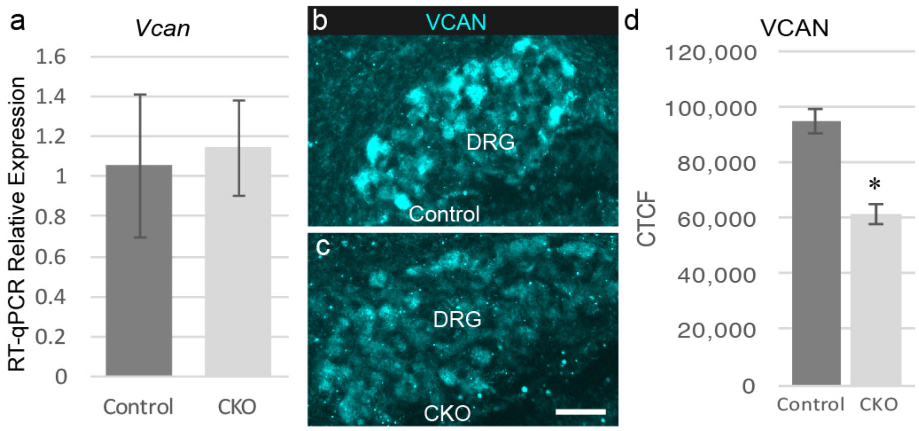


**Supplementary Figure 2**. Depleted levels of VCAN in Elongator CKO embryos. a) RT-qPCR confirms normal levels of *Vcan* transcript ($P$=0.78). b-d) Quantitative immunohistochemistry confirms decreased levels of VCAN protein ($P$<0.001). Error bars denote SD in a, and SEM in d. Scale bar: 40um

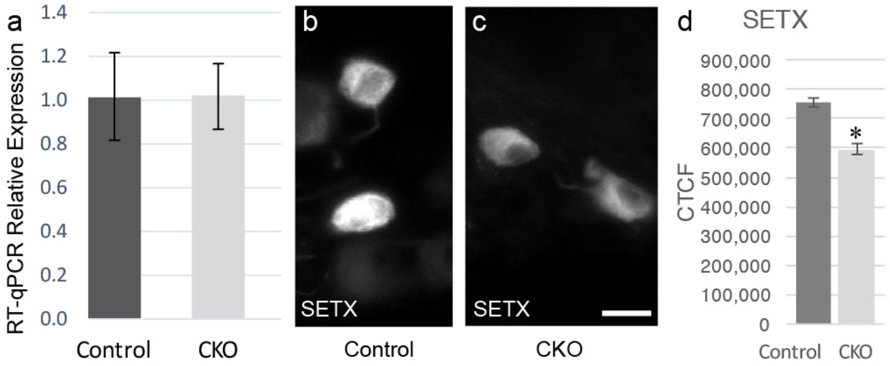


**Supplementary Figure 3**. Depleted levels of SETX in Elongator CKO embryos. a) RT-qPCR confirms normal levels of *Setx* transcript (*P*=1.0). b-d) Quantitative immunohistochemistry confirms decreased levels of SETX protein (*P*<0.001). Error bars denote SD in a, and SEM in d. Scale bar: 8um.

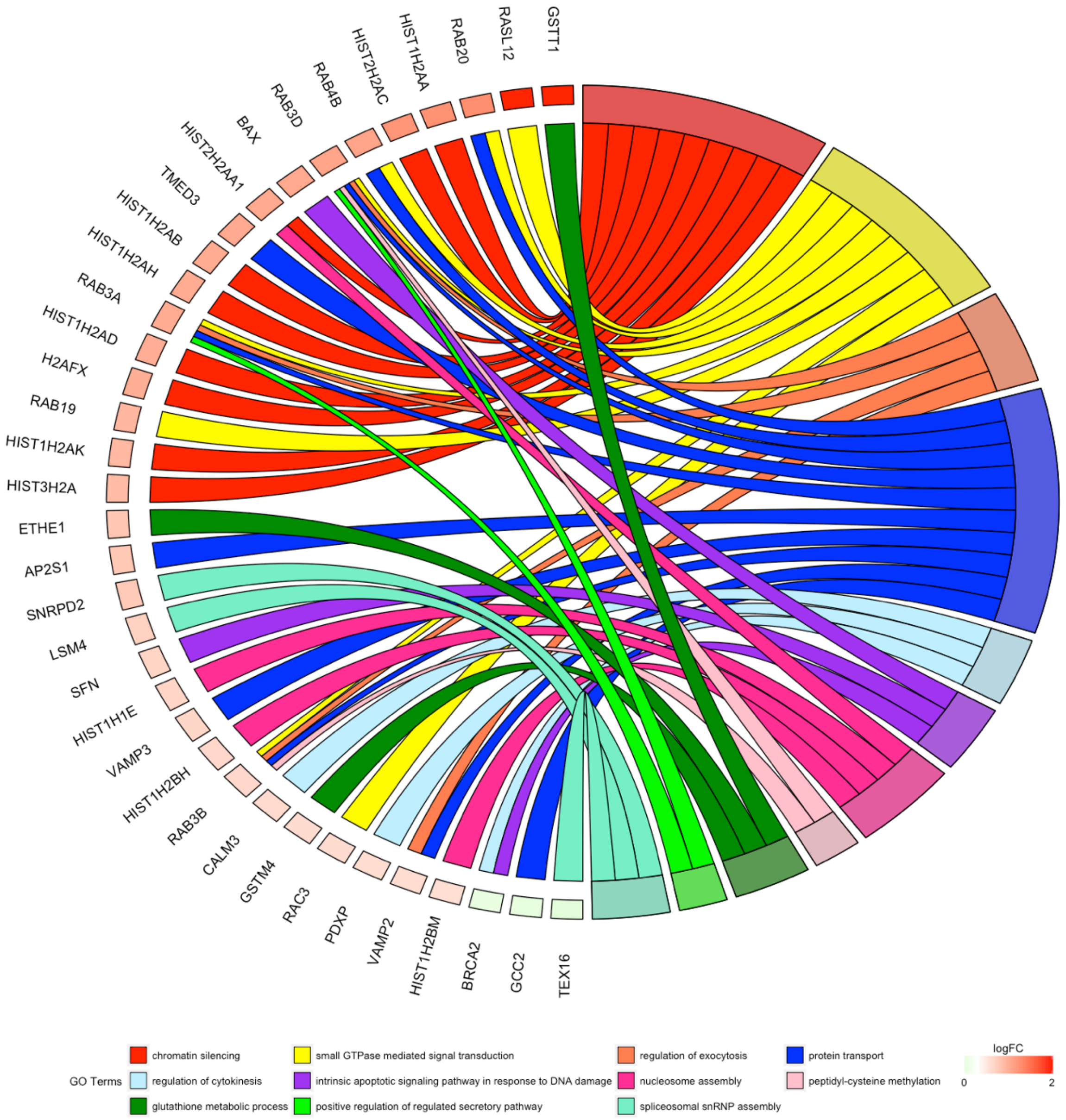


**Supplementary Figure 4.** Selected, significantly enriched GO biological processes for genes that are strong candidates for Elongator regulation (downregulated proteins encoded by genes with $\geq$ 1005 codons and an AA/AG ratio > 1.3 and upregulated proteins encoded by genes with $\leq$ 300 codons and an AA/AG ratio < 0.3). Proteins are arranged by decreasing log10 fold change from top to bottom. Ribbons connect GO terms to their associated proteins. *All genes were transcribed normally.
