# Supplementary material for: Elongator and codon bias regulate protein levels in mammalian peripheral neurons
Source: Nat Commun. 2018 Mar 1;9:889. doi: 10.1038/s41467-018-03221-z (PMC5832791; doi:10.1038/s41467-018-03221-z)
Supplement: Supplementary file 3 — Description of Additional Supplementary Files [file 41467_2018_3221_MOESM3_ESM.pdf]

Description of Additional Supplementary Files

File Name: Supplementary Data 1
Description: Proteome data for all normally transcribed genes (transcript fold change <1.5 and > -1.5) and codon usage for Lys, Gln, and Glu.

File Name: Supplementary Data 2
Description: Numerical data for Fig. 3b-e.

File Name: Supplementary Data 3
Description: List of genes within the mouse ORFeome that meet stringent indicators of Elongator dependence (≥ 1755 codons with AA:AG>1.3 or ≤ 150 codons with AA:AG <0.3).

File Name: Supplementary Data 4
Description: List of genes within the human ORFeome that meet less stringent indicators of Elongator dependence (≥ 1005 codons with AA:AG>1.3 or ≤ 150 codons with AA:AG <0.3).

File Name: Supplementary Data 5
Description: Potential Elongator targets: genes that were transcribed normally (transcript fold change < 1.5 and > -1.5) and consist of ≥ 1755 total codons with an AA:AG ratio > 1.3.

File Name: Supplementary Data 6
Description: Misregulated double-strand break repair genes (protein fold change ≤ -2.0, or ≥ 2.0).

File Name: Supplementary Data 7
Description: Codon usage of genes involved in double-strand break repair via nonhomologous end joining (NHEJ).

File Name: Supplementary Data 8
Description: Potential Elongator targets: genes that were transcribed normally (transcript fold change < 1.5 and > -1.5) and consist of ≤ 150 total codons with an AA:AG ratio < 0.2.

File Name: Supplementary Data 9
Description: List of upregulated genes that were used to determine GO biological processes: genes encoding proteins with a fold change ≥ 2 and an AA:AG ratio < 0.3 and transcript size ≤ 300 codons.
